# Understanding Organic Food Purchase Intention and Adoption in Türkiye: The Roles of Health, Sustainability, Product Information, and Social Media

**DOI:** 10.3390/foods15183356

**Published:** 2026-09-21

**Authors:** Ahmet Aslan

**Affiliations:** Department of Agricultural Economics, Malatya Turgut Özal University, Malatya 44210, Türkiye; ahmet.aslan@ozal.edu.tr

**Keywords:** organic food consumption, purchase intention, self-reported adoption, food consumer behavior, packaging informativeness, social media engagement

## Abstract

Organic food consumption has attracted increasing attention as consumers become more concerned about health, sustainability, product quality, and the reliability of food information. However, positive attitudes toward organic food do not necessarily translate into regular purchasing behaviour. This study investigates the factors associated with organic food purchase intention and self-reported adoption among consumers in Türkiye. Data were collected through an online survey from 893 consumers and analysed using partial least squares structural equation modelling (PLS-SEM). The model considers health concerns, personal image motivation, sustainable consumption orientation, content and naturalness expectations, packaging informativeness, and social media engagement as potential determinants of purchase intention and adoption. The findings support H1: all six antecedents were positively associated with purchase intention, with health concerns showing the strongest association (β = 0.225), followed by sustainable consumption orientation (β = 0.199). H2 was also supported: purchase intention was the strongest predictor of self-reported organic food adoption (β = 0.578), while personal image motivation and social media engagement showed significant direct associations. The mediation analysis further indicated that health concerns, sustainability orientation, content and naturalness expectations, and packaging informativeness were associated with adoption through purchase intention. These findings indicate that organic food adoption in Türkiye is associated with health and sustainability motives, product information, symbolic motivations, and digital engagement.

## 1. Introduction

Organic food consumption has become an important topic in food consumer behaviour because it reflects the interaction of health concern, perceived naturalness, sustainability orientation, product information, trust, and social influence. Consumers’ evaluations of organic foods are shaped not only by price and availability but also by perceptions of product purity, reduced chemical residue risk, certified production, credible labelling, and traceability [1,2,3,4,5]. Concerns about pesticide exposure, chemical residues, and food safety have further strengthened consumers’ interest in products perceived as safer and more natural [6,7,8]. Consumers’ willingness to pay for certified, traceable, or transparently labelled products also indicates that organic food choice is closely linked to trust and information-based assurance mechanisms [9,10,11,12].

Although organic food consumption is frequently discussed within the broader context of sustainable consumption, it should not be treated as equivalent to sustainable food behaviour in all respects. Organic food represents a specific food market context in which health, environmental concern, ethical values, product information, and consumer trust interact. The growing importance of sustainable food labelling and responsible consumption debates has increased interest in how consumers evaluate organic products and how these evaluations influence purchase intention and self-reported adoption [13,14,15]. In this context, health, safety, environmental awareness, ethical considerations, store trust, perceived retailer–product fit, and in-store experience are important antecedents of organic food choice and buying behaviour [16,17,18,19,20,21,22].

Digital environments add another layer to organic food decision-making by shaping how consumers access, interpret, and verify food-related information. Social media platforms, online reviews, influencer content, electronic word of mouth, and retailer websites function as food information channels through which consumers assess credibility, trust, risk, and product value [23,24,25,26]. However, misinformation and low-quality content may weaken trust in organic food claims, making credibility verification, trustworthy information architecture, multiple-source confirmation, community interaction, digital literacy, and information quality important for consumer confidence in organic food markets [27,28,29,30,31,32,33,34,35,36,37,38].

Türkiye is one of the leading agricultural producers in the Mediterranean region and has experienced steady growth in certified organic farming over the past two decades [39]. At the global level, organic agriculture and the trade in organic products have also expanded considerably in recent years. According to the Research Institute of Organic Agriculture [40], organic farming was practiced in 188 countries in 2022, covering a total of 96.4 million hectares, equivalent to approximately 2% of the world’s agricultural land. The geographical distribution of organic farmland indicates that 55% is located in Oceania, 18% in Europe, 10% in Latin America, 9.2% in Asia, 3.8% in North America, and 2.8% in Africa [40].

In terms of the total area under organic cultivation, Türkiye ranks 18th worldwide. With respect to the number of certified organic producers, it ranks 15th globally and fifth in Europe, highlighting the country’s important position in both the global and regional organic agriculture sector [40].

Despite this strong production capacity, the domestic organic food market in Türkiye remains relatively underdeveloped compared with regard to its export-oriented production structure. Previous studies have shown that although health consciousness and environmental awareness positively influence consumers’ intention to purchase organic food, high price premiums, limited product availability, information asymmetry, and a lack of trust in organic certification systems continue to constrain the development of the domestic market [41,42]. This imbalance is also reflected in per capita organic food consumption. While the highest annual per capita expenditures on organic products are observed in Switzerland (€437), Denmark (€365), Austria (€274), and Luxembourg (€259), annual per capita expenditure in Türkiye is only approximately €0.60, largely because domestic demand remains limited and production is primarily export-oriented [40].

Although consumers generally recognize the nutritional and environmental benefits of organic products, high prices remain one of the most significant structural barriers to organic food consumption [43]. Furthermore, uncertainty regarding the authenticity of organic products and the credibility of certification systems weakens consumer trust and hinders the translation of positive environmental attitudes into actual purchasing behaviour [44]. Studies conducted in the Eastern Mediterranean region have also shown that socio-demographic characteristics, particularly gender, influence organic food preferences, with female consumers generally exhibiting a stronger propensity to purchase organic products [45]. In addition, studies based on the Theory of Planned Behavior suggest that intrinsic motivations, such as health consciousness and personal well-being, interact with practical factors, including product availability and convenience, in shaping consumers’ organic food purchase decisions [46,47]. These findings are consistent with the broader international literature, which indicates that environmental awareness alone is insufficient to overcome the economic and structural barriers to organic food consumption in developing economies. Consequently, Türkiye provides an important research context for examining how psychological, economic, informational, and digital factors jointly shape organic food purchasing behaviour. Taken together, these findings underscore the importance of examining organic food consumption within the specific socio-economic context of Türkiye. They also highlight the need for an integrated framework that considers how psychological motivations, sustainability orientation, product-related information, packaging transparency, and digital information sources jointly influence consumers’ purchase intentions and subsequent self-reported adoption of organic food [43,46,47]. A central issue in organic food research is the gap between consumers’ stated intentions and their self-reported purchasing behaviour. Even when consumers express favourable attitudes toward organic products, routine adoption may be constrained by price, limited access, distrust in certification, convenience, social norms, cognitive dissonance, and other structural barriers [48,49,50,51]. This gap is particularly relevant among younger and sustainability-aware consumers, whose food choices may still be shaped by convenience, social expectations, and product accessibility [52]. Community-based consumption systems and credible product information may help reduce this intention–behaviour gap by strengthening trust and social support [53,54]. Social media may further reinforce green consumption by shaping subjective norms and perceived green value, while environmental knowledge can contribute to consumers’ green purchase behaviour [55,56].

Against this background, the present study examines how psychological, cognitive, and information-related factors are associated with organic food purchase intention and self-reported adoption in Türkiye. Specifically, the study integrates health concern, personal image motivation, sustainability orientation, content and naturalness expectations, packaging transparency, and social media engagement into a single food consumer behaviour model. The study contributes to the organic food consumer behaviour and food marketing literature by providing empirical evidence from Türkiye, examining purchase intention as a central link between antecedents and self-reported adoption, and distinguishing between central consumer motivations and supportive informational or social cues. In doing so, the study offers a food consumer-centred explanation of organic food adoption without treating organic food consumption as equivalent to sustainable food behaviour as a whole.

### 1.1. Conceptual Framework

Organic food consumption can be understood as a multidimensional food consumer behaviour process shaped by health-related motives, product evaluation, information-based trust, and digital food information environments. In organic food markets, consumers often associate organic products with lower exposure to pesticides, chemical residues, and food safety risks [7,8,17]. These perceptions make health concern an important antecedent of organic food purchase intention. Sustainability orientation may also contribute to intention because some consumers perceive organic products as compatible with ecological values, ethical consumption, and responsible lifestyles [50].

Product-related evaluations are also central to organic food choice. Expectations regarding product content, naturalness, additive absence, and minimal processing can shape perceived quality and trust. Natural ingredients, transparent production information, and clean-label characteristics may reduce uncertainty and strengthen purchase intention [2,48]. Similarly, packaging transparency, certification logos, traceability information, and informative labels operate as external food information cues that help consumers evaluate credibility and reduce information asymmetry [1,3,9]. Recent experimental evidence further indicates that the visual boundaries used to organise organic food information can influence perceived healthiness and purchase intention [57].

Digital environments may influence organic food consumption through informational and social mechanisms. Social media content, influencer posts, e-WOM, and user experiences may strengthen intention by increasing exposure to organic food information and making health- or sustainability-oriented food practices more visible [35,36,37,54]. However, misinformation may create distrust or cognitive dissonance when consumers encounter conflicting claims about organic products [38]. Therefore, reliable digital food information may support both intention formation and self-reported organic food adoption. Related studies of organic-product social media, virtual-influencer marketing, TikTok marketing, and AI-supported food recommendations also show that digital engagement, source credibility, informativeness, and transparency can shape purchase intention [58,59,60,61].

Within this framework, the study proposes that organic food purchase intention is shaped by health concerns, personal image motivation, sustainability orientation, content and naturalness expectations, packaging transparency, and social media engagement. Self-reported adoption is expected to be influenced by purchase intention and by selected image-related and digital factors. Purchase intention is also expected to mediate several antecedent–adoption relationships.

### 1.2. Theoretical Integration and Hypotheses

The model is anchored primarily in the Theory of Planned Behavior, which treats purchase intention as the most immediate motivational precursor of behaviour. In the organic food context, health concerns, sustainability orientation, content and naturalness expectations, and packaging informativeness represent beliefs and evaluations that can strengthen intention. Personal image motivation reflects perceived social approval and identity expression, while social media engagement provides informational and normative cues. Social identity and digital information mechanisms therefore complement the intention-based framework rather than constitute separate overarching theories [23,24,25,46,47,49,50].

Health concerns reflect beliefs about safety, pesticide residues, and personal well-being [6,7,8,16,17]. Sustainability orientation captures environmental and ethical evaluations [14,15,18,19]. Content and naturalness expectations and packaging informativeness represent product-level beliefs about purity, certification, traceability, and information credibility [1,2,3,4,9,10,48]. Personal image motivation captures the symbolic and socially visible meanings of organic consumption, and social media engagement represents exposure to electronic word of mouth, reviews, and credibility cues [23,24,25,34,36,37]. These mechanisms lead to the first comprehensive hypothesis:

**H1.**  *Health concerns, personal image motivation, sustainable consumption orientation, content and naturalness expectations, packaging informativeness and transparency, and social media engagement are positively associated with organic food purchase intention.*

Purchase intention should be the most immediate predictor of self-reported adoption. Personal image motivation and social media engagement may also have direct associations with adoption because identity expression, peer experiences, and digital information can accompany reported purchasing behaviour [29,33,34,36,37,38,49,50,52,54,62]. Accordingly:

**H2.**  *Purchase intention, personal image motivation, and social media engagement are positively associated with self-reported organic food adoption.*

The intention-based framework also implies that health, sustainability, content, and packaging evaluations may be associated with adoption indirectly by first strengthening purchase intention. These antecedents represent beliefs and product evaluations that precede a stated behavioural tendency [2,3,6,7,8,14,15,18,48]. Therefore:

**H3.**  *Purchase intention mediates the relationships of health concerns, sustainable consumption orientation, content and naturalness expectations, and packaging informativeness and transparency with self-reported organic food adoption.*

**Figure 1:**
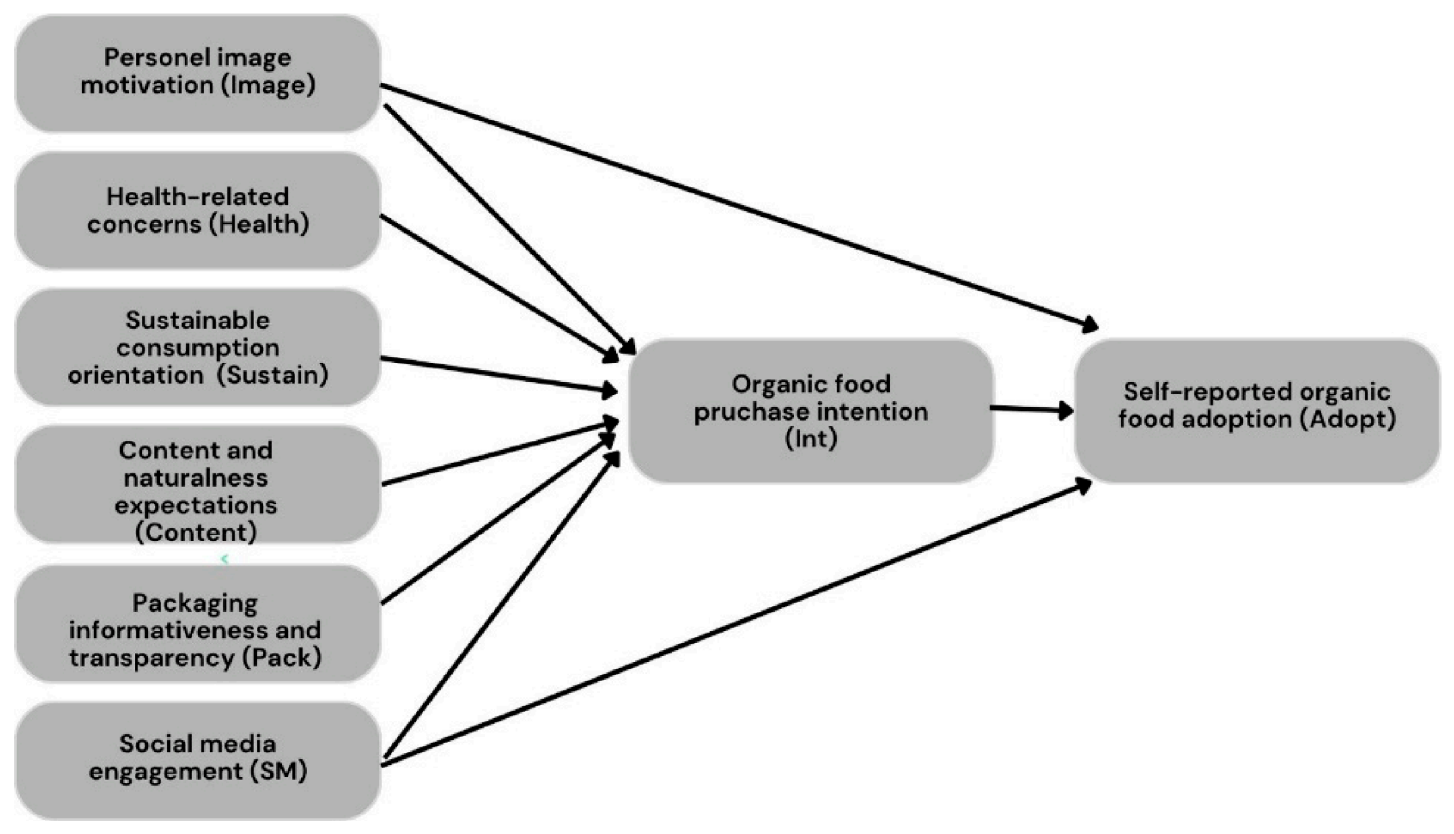
Proposed Research Model.

Figure 1 presents the proposed research model developed for this study. Based on the relevant literature, the model illustrates the hypothesised relationships between the proposed antecedents of organic food purchase intention and self-reported organic food adoption, including the mediating role of purchase intention.

## 2. Materials and Methods

### 2.1. Research Design and Data Collection

This study employed a quantitative, cross-sectional survey design to examine the cognitive, informational, motivational, and digital factors associated with organic food purchase intention and self-reported organic food adoption. Data were collected in Türkiye through an online questionnaire administered via Google Forms in 2026. After data screening, 893 valid responses were retained for analysis.

The questionnaire consisted of measurement items adapted from the organic food consumption and food consumer behaviour literature and administered in Turkish using a 5-point Likert-type scale. Appendix A reports the original Turkish wording and an English translation of the 32 indicators used in the reported measurement and structural models. The adoption items were adapted from [63], and the remaining scales were adapted from [46,64,65,66,67,68,69].

### 2.2. Measures

The study model included eight latent constructs. Health concern captured perceived safety and personal health benefits. Personal image motivation represented symbolic, status-related, and socially responsible meanings attached to organic food choice. Sustainable consumption orientation reflected environmental and production-related beliefs. Content and naturalness expectations captured preferences for natural colour and the absence of preservatives and artificial colourants. Packaging informativeness and transparency represented certification and producer information available at the point of purchase. Social media engagement captured trust in, behavioural responses to, and active checking of organic food comments online. Purchase intention represented favourable evaluation and willingness to buy, while self-reported adoption represented stated preference, trial, and continued purchasing behaviour. These operational definitions clarify the distinct conceptual role of each construct in the model.

### 2.3. Data Analysis

The data were analysed using partial least squares structural equation modelling (PLS-SEM) with SmartPLS 4. PLS-SEM was selected because the study examines a multidimensional explanatory model with several antecedents of purchase intention, direct predictors of self-reported adoption, and indirect effects through purchase intention.

The measurement model was assessed using outer loadings, Cronbach’s alpha, composite reliability (CR), and average variance extracted (AVE). Discriminant validity was evaluated using the Fornell–Larcker criterion and the heterotrait–monotrait ratio (HTMT). The structural model was examined using path coefficients, *t*-values, *p*-values, explained variance (R^2^), effect sizes (f^2^), and predictive relevance (Q^2^). Direct and indirect effects were tested using bootstrapping with 5000 resamples. Model fit was evaluated using SRMR, d_ULS, d_G, chi-square, and NFI, while recognising that global fit indices in PLS-SEM should be interpreted as supplementary rather than definitive evidence.

### 2.4. Assessment of Common Method Bias

Because the study relies on single-source, self-reported, cross-sectional survey data, common method bias (CMB) was addressed through procedural and statistical checks. Procedurally, the survey was anonymous and voluntary, respondents were informed that there were no right or wrong answers, and items representing distinct constructs were conceptually separated.

Statistically, three diagnostic procedures were applied. Harman’s single-factor test showed that the first unrotated factor accounted for 38.17% of the total variance, below the commonly used 50% threshold [70]. Full-collinearity VIF values ranged from 1.63 to 3.00, below the conservative 3.3 threshold proposed in [71]. Inner VIF values ranged from 1.57 to 2.09 for the purchase-intention equation and from 1.03 to 1.77 for the adoption equation, below the 3.0 and 5.0 thresholds discussed in the PLS-SEM literature [72]. These diagnostics do not demonstrate the absence of common method bias. Harman’s test has limited sensitivity because it relies on a single exploratory factor solution, and full-collinearity VIF is an indirect diagnostic that may not detect every method effect. The results therefore indicate only that no dominant single factor or severe collinearity pattern was detected. Residual bias remains possible because all variables were collected from the same respondents at one time using self-reports.

### 2.5. Control Variables and Exploratory Sensitivity Analysis

An exploratory control-adjusted sensitivity analysis was conducted to assess whether the main structural relationships changed after demographic controls were introduced. The dataset included age, gender, and monthly household income. Education was not collected and therefore could not be included as a control variable.

Age was treated as continuous. Two entries that appeared to contain birth years were corrected by subtracting them from 2025, and 11 responses with ages below 18 were excluded. Gender was used only in the exploratory control-adjusted analysis and was dummy-coded as male = 0 and female = 1. Ten respondents who preferred not to disclose their gender remained in the baseline PLS-SEM sample but were excluded from this supplementary regression because their responses could not be assigned to either category of the binary control variable and the group was too small to estimate a separate category reliably. This treatment may reduce inclusiveness and introduce selection bias, so the gender-control results should be interpreted cautiously. Income was retained as a continuous variable measured in Turkish Lira, and a supplementary robustness check using log-transformed income was also conducted.

After these adjustments, the exploratory control-adjusted analysis was based on 869 observations, compared with 893 observations in the baseline PLS-SEM model. Because estimation of single-item controls in the primary PLS-SEM specification was not feasible in the analytical environment used for this study, composite-score ordinary least squares regression was used as a supplementary sensitivity check. Each latent construct was represented by the arithmetic mean of its items, and age, gender, and income were entered as controls. This exploratory analysis does not replace the primary PLS-SEM model or provide an equivalent test of the latent-variable relationships. Detailed estimates are reported in Appendix B.

## 3. Results

### 3.1. Measurement Model

The measurement model was assessed in terms of indicator reliability, internal consistency, convergent validity, and discriminant validity. Outer loadings ranged from 0.768 to 0.908, exceeding the recommended 0.70 threshold and indicating adequate indicator reliability (Table 1).

Internal consistency was satisfactory. Cronbach’s α values ranged from 0.791 to 0.933, while composite reliability (CR) values ranged from 0.801 to 0.936. All values were above the recommended 0.70 threshold. Convergent validity was also supported, as average variance extracted (AVE) values ranged from 0.664 to 0.774, exceeding the 0.50 threshold for all constructs. Overall, the measurement model provided an adequate basis for testing the structural relationships.

### 3.2. Discriminant Validity

Discriminant validity was evaluated using the Fornell–Larcker criterion and HTMT ratios (Table 2). In the Fornell–Larcker matrix, the square root of the AVE for each construct was higher than its correlations with other constructs, indicating that each construct shared more variance with its own indicators than with other constructs.

The HTMT values were also below the 0.85 threshold. The highest HTMT value was observed between purchase intention and self-reported organic food adoption (0.830), which is theoretically expected given the close relationship between intention and food purchasing-related outcomes. However, this value remained below the recommended threshold, supporting discriminant validity.

### 3.3. Common Method Bias and Collinearity Diagnostics

Common method and collinearity diagnostics were examined before the structural model was interpreted (Table 3). The first unrotated factor explained 38.17% of the total variance, and full-collinearity VIF values ranged from 1.63 to 3.00. Inner VIF values ranged from 1.57 to 2.09 for the purchase-intention equation and from 1.03 to 1.77 for the adoption equation. No dominant single factor or severe collinearity pattern was detected. These diagnostics do not rule out common method bias, particularly given the cross-sectional, single-source, and self-reported data.

### 3.4. Model Fit

The model fit indices suggest an acceptable level of fit for the PLS-SEM model. The SRMR value was 0.076, below the commonly used 0.08 threshold. The NFI value was 0.812, indicating an adequate basic fit. The d_ULS, d_G, and chi-square values are reported for completeness and should be interpreted cautiously in PLS-SEM (Table 4).

### 3.5. Structural Model Results

The structural model results are presented in Table 5. The model explained 47.6% of the variance in organic food purchase intention and 67.7% of the variance in self-reported organic food adoption.

All six dimensions specified in H1 were statistically significant and positive. Health-related concerns, sustainable consumption orientation, personal image motivation, packaging transparency, content and naturalness expectations, and social media engagement were positively associated with purchase intention. Health-related concerns and sustainable consumption orientation showed the strongest associations, so H1 was supported across all proposed antecedents.

The three predictors specified in H2—personal image motivation, purchase intention, and social media engagement—were positively associated with self-reported organic food adoption. Purchase intention was the strongest predictor. Effect-size results indicate that several paths had small effects, whereas the purchase intention–adoption path had the largest effect in the model (f^2^ = 0.748). H2 was therefore supported across all three proposed relationships.

### 3.6. Indirect Effects

The indirect effect results are presented in Table 6. Purchase intention significantly mediated the relationships between health concern, sustainability orientation, content and naturalness expectations, packaging transparency, and self-reported organic food adoption.

The indirect associations specified in H3 were statistically significant for health concern, sustainability orientation, content and naturalness expectations, and packaging transparency. H3 was therefore supported across all four antecedents. The results indicate that these evaluations were associated with self-reported adoption through their relationships with purchase intention.

### 3.7. Exploratory Control-Adjusted Sensitivity Analysis

An exploratory control-adjusted composite-score regression was conducted to assess whether the direction and statistical significance of the principal relationships changed after demographic controls were introduced. Because this analysis uses observed composite scores and a smaller analytic sample, it is treated as a sensitivity check rather than a robustness confirmation. Detailed coefficients are provided in Appendix B.

Appendix B reports the control-adjusted composite-score regression estimates.

The exploratory estimates preserved the direction and statistical significance of the principal paths. This consistency is reassuring, but it does not eliminate concerns arising from composite-score estimation, the reduced sample, or omitted demographic characteristics.

Age showed a small negative association with self-reported adoption, while gender and income were not statistically significant. These coefficients should be interpreted cautiously because the analysis was exploratory, education was unavailable, and respondents who did not disclose gender could not be represented in the binary control variable.

## 4. Discussion

The findings support an intention-based explanation of organic food adoption in Türkiye. Health concerns, sustainability orientation, personal image motivation, packaging informativeness, content and naturalness expectations, and social media engagement were positively associated with purchase intention, while intention had the strongest association with self-reported adoption. This pattern is consistent with the Theory of Planned Behavior: consumers’ beliefs and evaluations are associated with adoption mainly through a proximal behavioural intention. The results should nevertheless be interpreted as associations because the cross-sectional design cannot establish temporal or causal order.

Health concern and sustainability orientation had the strongest associations with purchase intention among the six antecedents. In Türkiye, these motives operate within a market characterised by very low per capita organic expenditure, export-oriented production, price premiums, uneven availability, and continuing doubts about certification [40,41,42,43,44,45,46,47]. Health and environmental values may therefore create favourable intentions without overcoming affordability, access, and trust barriers. The strong intention–adoption path does not show that these structural barriers are unimportant; it indicates that respondents reporting stronger intentions also reported greater adoption.

Packaging informativeness and content and naturalness expectations were positively associated with intention. Certification marks, producer details, traceability information, natural colour, and the absence of preservatives or artificial colourants can help consumers evaluate claims that are otherwise difficult to verify [73,74,75,76,77,78,79,80,81,82,83,84,85,86,87]. In the Turkish market, consistent information across labels, retail channels, and digital platforms may be particularly important because limited familiarity and distrust can intensify information asymmetry. These results support clearer and verifiable communication, but they do not establish that adding more claims to a package will cause purchases.

Personal image motivation was associated with both intention and self-reported adoption, but its effects were smaller than the intention–adoption relationship. The finding may reflect identity expression, perceived social approval, or socially desirable responding rather than a stable status motive. Organic food choices can signal health consciousness and environmental awareness, especially in socially visible settings, yet the construct combines responsibility, popularity, and status meanings. The result should therefore not be interpreted as evidence that conspicuous consumption is the principal reason for organic purchasing.

Social media engagement also showed positive but comparatively small associations with intention and adoption. Online comments may provide product information, peer experience, and reassurance; they may also transmit misinformation, promotional content, and selective positive experiences [23,24,25,26,27,28,29,30,31,32,33,34,35,36,37,38]. The measure captures trust in comments, checking behaviour, and responses to positive and negative reviews, so the observed association does not isolate a single platform mechanism. Digital communication should consequently prioritise verifiable certification and producer information rather than relying only on influencer visibility or favourable comments.

Sensory quality is another relevant part of the purchase decision. Taste, aroma, texture, colour, freshness, and visual appearance can determine whether an initially motivated consumer accepts and repeatedly purchases an organic food. The present survey captured one retained health item referring to taste and content-related expectations concerning natural colour, but it did not include a dedicated multidimensional sensory-quality construct or a sensory evaluation. The model therefore cannot estimate the independent effect of sensory experience. Future studies should combine consumer surveys with tasting or product-evaluation designs to test whether sensory acceptance strengthens or weakens the transition from intention to repeat purchase [72].

The indirect effects further support the central role of intention: health concern, sustainability orientation, content expectations, and packaging transparency were associated with adoption through purchase intention. For Türkiye, this suggests that credible certification, understandable labelling, and trustworthy digital information may help consumers form intentions, but market development also depends on price and physical access. Producers, retailers, certification bodies, and public agencies should therefore treat information quality as one component of a broader market response rather than as a substitute for affordability and availability.

## 5. Conclusions

This study found that health concern, sustainability orientation, content and naturalness expectations, packaging informativeness, personal image motivation, and social media engagement were positively associated with organic food purchase intention in Türkiye. Purchase intention had the strongest association with self-reported adoption and mediated the relationships of health, sustainability, content, and packaging evaluations with adoption. The results support an intention-based account of organic food choice while showing that symbolic and digital factors also have smaller direct associations with reported adoption.

### 5.1. Practical Implications

The findings have direct implications for Türkiye’s domestic organic food market. Producers and retailers should present certification, producer identity, ingredients, and traceability information in clear and verifiable forms at both physical and digital points of sale. Certification bodies and public agencies can support purchase confidence through recognisable labels, accessible verification systems, and consumer education about permitted organic-production practices. Marketers should avoid treating favourable social-media attention as a substitute for evidence-based product information. Because intention alone may not overcome high prices and limited availability, market actors should also improve distribution and communicate value without overstating health or environmental claims. Product development and repeat-purchase strategies should consider taste, aroma, texture, freshness, and appearance alongside certification and naturalness.

### 5.2. Limitations and Future Research

Several limitations define the scope of the conclusions. The cross-sectional design and self-reported measures do not establish causality or observed purchasing behaviour. The online sample may underrepresent consumers with limited digital access and does not support unrestricted generalisation to all consumers or regions of Türkiye. Harman’s single-factor test and VIF diagnostics cannot rule out common method bias. Education was not collected, and the binary coding used in the exploratory control analysis excluded respondents who did not disclose gender. The personal-image and social-media constructs also combine multiple symbolic and digital mechanisms that could be separated in future research.

Future studies should use longitudinal, experimental, or observed-purchase designs; compare regions, income groups, retail channels, and product categories; and test price, availability, and certification trust as moderators of the intention–behaviour relationship. Dedicated sensory measures and controlled tasting studies should examine taste, aroma, texture, colour, freshness, and appearance as determinants of initial choice and repeat purchase. Research should also distinguish social approval, status signalling, electronic word of mouth, influencer exposure, and verification behaviour to clarify why personal image and social media are associated with adoption.

## Figures and Tables

**Table 1:** Reliability and Convergent Validity Assessment.

Construct/Item	Outer Loadings	Cronbach’s α	CR	AVE
Adopt1	0.888	0.903	0.906	0.774
Adopt2	0.895			
Adopt3	0.868			
Adopt4	0.868			
Content2	0.809	0.799	0.808	0.71
Content3	0.865			
Content4	0.853			
Health1	0.811	0.791	0.801	0.704
Health2	0.874			
Health3	0.831			
INT1	0.78	0.933	0.936	0.75
INT2	0.888			
INT3	0.906			
INT4	0.908			
INT5	0.878			
INT6	0.829			
Image1	0.821	0.834	0.846	0.664
Image2	0.793			
Image3	0.833			
Image4	0.812			
Pack1	0.824	0.877	0.884	0.73
Pack2	0.888			
Pack3	0.823			
Pack4	0.881			
SM1	0.878	0.888	0.908	0.747
SM2	0.908			
SM3	0.816			
SM4	0.852			
Sustain1	0.78	0.839	0.845	0.674
Sustain2	0.768			
Sustain3	0.867			
Sustain4	0.866			

Source: Authors’ calculations based on survey data.

**Table 2:** Convergent and Discriminant Validity.

**Fornell–Larcker**								
	**Adopt**	**Content**	**Health**	**Int**	**Image**	**Pack**	**SM**	**Sustain**
Adopt	0.88							
Content	0.327	0.843						
Health	0.581	0.325	0.839					
Int	0.77	0.43	0.557	0.866				
Image	0.64	0.237	0.613	0.528	0.815			
Pack	0.252	0.636	0.209	0.368	0.135	0.855		
SM	0.499	0.145	0.423	0.395	0.573	0.035	0.864	
Sustain	0.543	0.376	0.57	0.557	0.578	0.286	0.413	0.821
**HTMT**								
	**Adopt**	**Content**	**Health**	**Int**	**Image**	**Pack**	**SM**	**Sustain**
Adopt								
Content	0.365							
Health	0.674	0.397						
Int	0.83	0.491	0.641					
Image	0.725	0.283	0.724	0.573				
Pack	0.272	0.76	0.25	0.407	0.168			
SM	0.553	0.148	0.489	0.423	0.678	0.056		
Sustain	0.615	0.437	0.693	0.626	0.665	0.326	0.472	

Source: Authors’ calculations based on survey data.

**Table 3:** Results of Common Method Bias and Collinearity Diagnostics.

Diagnostic Test	Criterion	Result	Interpretation
Harman’s single-factor test	First unrotated factor < 50% of total variance	38.17%	No dominant single factor detected
Full collinearity VIF	All construct VIFs < 3.3	1.63–3.00	Below the stated threshold; CMB not ruled out
Inner VIF, INT equation	All predictor VIFs < 3.0/5.0	1.57–2.09	No critical multicollinearity
Inner VIF, Adopt equation	All predictor VIFs < 3.0/5.0	1.03–1.77	No critical multicollinearity

Note. Harman’s single-factor test was conducted using exploratory principal component analysis on all 32 manifest indicators. Full collinearity VIF was estimated by regressing each construct on all remaining constructs. Inner VIF values were computed within each structural equation separately. Source: Authors’ calculations based on survey data.

**Table 4:** Structural Equation Model Fit Indices.

	SRMR	d_ULS	d_G	Chi-Square	NFI
Model	0.076	3.015	0.712	3967.1	0.812

Source: Authors’ calculations based on survey data.

**Table 5:** Path Coefficients and Explained Variance in the Structural Model.

Hypotheses	Direct Effect	β	*t*	*p* Values	f^2^	R^2^	Decision
H1	Health → Int	0.225	5.767	0.001	0.052		Supported
H1	Image → Int	0.166	4.321	0.001	0.025		Supported
H1	Sustain → Int	0.199	5.016	0.001	0.041	0.476	Supported
H1	Content → Int	0.13	3.544	0.001	0.017		Supported
H1	Pack → Int	0.157	3.722	0.001	0.027		Supported
H1	SM → Int	0.098	2.607	0.009	0.012		Supported
H2	Image → Adopt	0.267	8.784	0.001	0.122		Supported
H2	Int → Adopt	0.578	19.502	0.001	0.748	0.677	Supported
H2	SM → Adopt	0.121	4.2	0.001	0.028		Supported

Source: Authors’ calculations based on survey data.

**Table 6:** Indirect Effects Through Purchase Intention.

Hypotheses	Indirect Effect	β	*t*	*p* Values	Decision
H3	Health → Int → Adopt	0.13	5.207	0.001	Supported
H3	Sustain → Int → Adopt	0.115	4.786	0.001	Supported
H3	Content → Int → Adopt	0.075	3.439	0.001	Supported
H3	Pack → Int → Adopt	0.091	3.764	0.001	Supported

Source: Authors’ calculations based on survey data.

## Data Availability

The original contributions presented in the study are included in the article, further inquiries can be directed to the corresponding author.

## Appendix A. Questionnaire Items Used in the Model

**Table A1:** English wording of the 32 indicators used in the reported measurement and structural models.

Code	Construct	English Translation
Health1	Health Concern	Organic foods can be consumed safely.
Health2	Health Concern	Organic food tastes good.
Health3	Health Concern	I consume organic foods to maintain my physical fitness.
Image1	Personal Image Motivation	I prefer organic foods because of my environmental awareness.
Image2	Personal Image Motivation	I prefer organic foods because they are a popular dietary trend.
Image3	Personal Image Motivation	I prefer organic foods because they are a status symbol for me.
Image4	Personal Image Motivation	I prefer organic foods because of my sense of responsibility.
Sustain1	Sustainable Consumption Orientation	Consuming organic food reinforces my environmental awareness.
Sustain2	Sustainable Consumption Orientation	I prefer organic foods because pesticides permitted for organic production are used in their cultivation.
Sustain3	Sustainable Consumption Orientation	I like organic foods because chemical-free fertilisers are used in their cultivation.
Sustain4	Sustainable Consumption Orientation	Organic foods are processed in an environmentally friendly manner.
Content2	Content and Naturalness Expectations	I want organic foods to have natural colours.
Content3	Content and Naturalness Expectations	Organic foods should not contain preservatives.
Content4	Content and Naturalness Expectations	Organic foods should not contain artificial colourants.
Pack1	Packaging Informativeness and Transparency	Organic ingredients should be stated on organic food packaging.
Pack2	Packaging Informativeness and Transparency	Organic food packaging must display an organic certificate.
Pack3	Packaging Informativeness and Transparency	Organic food packaging should include producer information.
Pack4	Packaging Informativeness and Transparency	Organic information should appear on the packaging.
SM1	Social Media Engagement	I trust comments about organic foods on social media.
SM2	Social Media Engagement	A positive comment on social media encourages me to buy an organic food.
SM3	Social Media Engagement	A negative comment on social media discourages me from buying organic food.
SM4	Social Media Engagement	I check comments on social media before buying organic food.
INT1	Purchase Intention	I have a positive view of organic foods.
INT2	Purchase Intention	I have a strong tendency to purchase organic foods.
INT3	Purchase Intention	I include organic food among my purchasing preferences.
INT4	Purchase Intention	I have a strong intention to purchase organic food.
INT5	Purchase Intention	When buying food, my first consideration is to purchase organic food.
INT6	Purchase Intention	My evaluation of organic foods is positive.
Adopt1	Self-Reported Organic Food Adoption	I prefer to purchase organic foods.
Adopt2	Self-Reported Organic Food Adoption	Organic food products are my solution for safe food consumption.
Adopt3	Self-Reported Organic Food Adoption	I try newly introduced organic foods when they first become available.
Adopt4	Self-Reported Organic Food Adoption	I continually purchase and use new organic foods.

## Appendix B. Exploratory Control-Adjusted Sensitivity Analysis

**Table A2:** Exploratory Control-Adjusted Composite-Score Regression with Demographic Controls.

Path	Baseline β (PLS)	Control-Adj. β (OLS)	*t*	*p*	f^2^	Interpretation
Health → Int	0.225	0.204	6.771	<0.001	0.053	Robust ***
Image → Int	0.166	0.109	4.035	<0.001	0.019	Robust ***
Sustain → Int	0.199	0.192	6.264	<0.001	0.046	Robust ***
Content → Int	0.13	0.179	4.167	<0.001	0.02	Robust ***
Pack → Int	0.157	0.219	4.754	<0.001	0.026	Robust ***
SM → Int	0.098	0.065	2.839	0.005	0.009	Robust **
Image → Adopt	0.267	0.23	9.845	<0.001	0.112	Robust ***
Int → Adopt	0.578	0.686	24.907	<0.001	0.72	Robust ***
SM → Adopt	0.121	0.097	4.475	<0.001	0.023	Robust ***
Age → Int	—	−0.001	−0.904	0.366	n/a	Non-significant
Gender → Int	—	0.045	1.125	0.261	n/a	Non-significant
Income → Int	—	<0.001	−1.208	0.227	n/a	Non-significant
Age → Adopt	—	−0.004	−2.838	0.005	n/a	Significant **
Gender → Adopt	—	−0.01	−0.27	0.787	n/a	Non-significant
Income → Adopt	—	<0.001	−0.235	0.814	n/a	Non-significant

Note. Baseline coefficients are from PLS-SEM (N = 893); control-adjusted estimates are from exploratory composite-score OLS regression with age, gender, and income controls (N = 869). Gender: 0 = male, 1 = female. ** *p* < 0.01; *** *p* < 0.001. Source: Author’s calculations based on survey data.
